# Mobility Deficits Assessed With Mobile Technology: What Can We Learn From Brain Iron-Altered Animal Models?

**DOI:** 10.3389/fneur.2019.00833

**Published:** 2019-08-08

**Authors:** Franziska Hopfner, Markus A. Hobert, Corina Maetzler, Clint Hansen, Minh Hoang Pham, Caroline Moreau, Daniela Berg, David Devos, Walter Maetzler, The Fair-Park II study group

**Affiliations:** ^1^Department of Neurology, University Hospital Schleswig-Holstein, Christian-Albrechts-Universität zu Kiel, Kiel, Germany; ^2^Department of Psychiatry and Psychotherapy, Ludwig-Maximilians-Universität München, Munich, Germany; ^3^Department of Movement Disorders and Neurology, Faculty of Medicine, Lille University Hospital, Lille University, INSERM U1171, Lille, France; ^4^Departments of Medical Pharmacology and Movement Disorders, Lille University Hospital, Lille University, INSERM U1171, Lille, France

**Keywords:** Parkinson's disease, restless legs syndrome, mobility assessment, iron, animal models

## Abstract

**Background:** Recent developments in mobile technology have enabled the investigation of human movements and mobility under natural conditions, i.e., in the home environment. Iron accumulation in the basal ganglia is deleterious in Parkinson's disease (i.e., iron accumulation with lower striatal level of dopamine). The effect of iron chelation (i.e., re-deployment of iron) in Parkinson's disease patients is currently tested in a large investigator-initiated multicenter study. Conversely, restless legs syndrome (RLS) is associated with iron depletion and higher striatal level of dopamine. To determine from animal models which movement and mobility parameters might be associated with iron content modulation and the potential effect of therapeutic chelation inhuman.

**Methods:** We recapitulated pathophysiological aspects of the association between iron, dopamine, and neuronal dysfunction and deterioration in the basal ganglia, and systematically searched PubMed to identify original articles reporting about quantitatively assessed mobility deficits in animal models of brain iron dyshomeostasis.

**Results:** We found six original studies using murine and fly models fulfilling the inclusion criteria. Especially postural and trunk stability were altered in animal models with iron overload. Animal models with lowered basal ganglia iron suffered from alterations in physical activity, mobility, and sleep fragmentation.

**Conclusion:** From preclinical investigations in the animal model, we can deduce that possibly also in humans with iron accumulation in the basal ganglia undergoing therapeutic chelation may primarily show changes in physical activity (such as daily “motor activity”), postural and trunk stability and sleep fragmentation. These changes can readily be monitored with currently available mobile technology.

## Background

Mobile technology (mainly reflected by “wearables”) is a dynamically developing field with relevant implications for the health and fitness sector, and also for medical routine and clinical trials (1). Wearables for the assessment of movements and mobility are microelectromechanical systems with multiple degrees of freedom (DOF; e.g., 3D accelerometers, 3D gyroscopes, and 3D magnetometers) (2, 3). They enable continuous assessment during 24 h over many days, and make possible data collection and evaluation of disease progression and response to treatment based on uninstructed, unsupervised, and unobserved movements (1, 3–5). It seems realistic that such data will soon be used as complementary information for treatment decisions in diseases associated with movement deficits, such as Parkinson's disease (PD) (6) and restless legs syndrome (RLS).

This development is, at least partly, driven by accumulating evidence that humans perform similar movements differently in the home environment and in the doctor's practice, hospital and research lab, especially with regard to daily-relevant movements (4, 7). Therefore, clinical assessment may not always reflect daily-relevant information for the affected. Moreover, physical activity and mobility can by definition only be evaluated in the natural environment of the user, and some movement abnormalities, such as those happening during sleep, may occur only in the “unobserved,” but not in the usual professional, environment (8). Specific algorithms for the detection of such movements in the natural environment are currently developed by us and other groups (9–12).

While the usage of mobile technology is widely applied in humans, animal research also benefits from the technological development in movement and mobility assessment, as evidenced by behavioral measures in scientifically used animal models, animal husbandry, or the wild (13, 14). So far translation of quantitative data of movement and mobility from animal models to human has not been performed but it seems likely that quantitatively assessed movement changes in animals correspond to human diseases. Therefore, they may inform about (corresponding) human behavior.

In this review we aim to transfer pathophysiology of disturbed brain iron homeostasis in phenomenology of established PD and RLS animal models (Tables 1, 2). Our approach is based on accumulating evidence that iron accumulation in the basal ganglia leads to a PD-like, hypokinetic movement deficit, and lack of iron may be associated with a certain level of hyperkinetic movements resembling RLS features. We will discuss animal models associated with iron overload (25) and iron deficiency (26). This discussion should help defining a set of promising parameters to monitor changes of movement and mobility in a currently built mobile technology-based dataset obtained from the natural environment of PD patients under investigational treatment with an iron chelator (FAIR PARK II study, www.fairpark2.eu). FAIR PARK II is an investigator-initiated trial testing the effects of a conservative iron chelation therapy -i.e., chelation and redeployment of iron to limit iron sequestration and iron deficit- on the progression of handicap in PD.

**Table 1:** Animal models for elevated brain iron.

**Author**	**Animal model**	**Iron changes**	**Movement / mobility outcome parameter**	**Result**	**Suggested relevance for human mobility and movements**
Vidal et al. (15)	Transgenic mouse of a human ferritin light polypeptide cDNA carrying a thymidine and cytidine insertion at position 498 (FTL498 499InsTC)	Iron overload in the putamen, globus pallidus, thalamus, cerebellum, hippocampus, and the olfactory bulb	(i) Latency to fall from the rotarod [accelerating rotarod (16)] (ii) Position reflexes during suspension of the mice by their tails (iii) grooming and incoordination	(i) Mutated mice showed shorter latency to fall from the rotarod (ii) Mutated mice showed flexion of front and hindlimbs inward, with paws clasping together and drawing in toward the body and also hunchbacked posture when walking, low body position with some degree of lordosis, and shuffling gait with dragging hindlimbs (iii) Mutated mice showed abnormal grooming and incoordination	Dynamic balance disturbance Reduction of position reflexes Posture deficits Reduced activity/apathy Impaired fine-motor dexterity and motor coordination
Levenson et al. (17)	Wild type mouse with (Fe-) and without dietary iron restriction (Fe+) treated with MPTP	MPTP leads to iron overload in TH-positive neurons (18)	Number of falls from the rotarod within 5 min (19)	MPTP-treated Fe+ mice (but not MPTP-treated Fe- mice) had higher number of falls from the rotarod and lower total amount of time spent on the rotarod	Dynamic balance disturbance
Zhu et al. (20)	Drosophila melanogaster with (Fe–) and without dietary iron restriction (Fe+) overexpressing A53T or A30P alpha-synuclein	Iron overload in dopaminergic neurons in mutated Fe+ animals	Climbing ability in a plastic column (Negative geotaxis assay)	Fe+ mutant flies showed decreasing geotaxis performance	Reduced motor activity, reduced amount of voluntary movements
Maccarinelli et al. (21)	Transgenic mouse of a human ferritin light polypeptide cDNA carrying a thymidine and cytidine insertion at position 498 (FTL498 499InsTC)	Iron overload in the putamen, globus pallidus, thalamus, cerebellum, hippocampus, and the olfactory bulb	Latency to fall from the rotarod before the end of the 5-min test session [accelerating rotarod (22)]	Both the mutation and age had a negative effect on the latency (i.e., shorter) to fall from the rotarod	Dynamic balance disturbance

**Table 2:** Animal models for reduced brain iron.

**Author**	**Animal model**	**Iron changes**	**Movement/mobility outcome parameter**	**Result**	**Suggested relevance for free human movement**
Freeman et al. (23)	Drosophila melanogaster with loss of functional BTBD9 (Drosophila homolog CG1826)	Iron depletion in dopaminergic neurons (BTBD9 modulates ferritin levels through reducing iron regulatory protein-2 levels)	Walking speed, total distance covered in 5 min, number of walks between two black bars, total walking time, number of breaks, average walking bout distance	Mutated flies showed increased total distance, increased number of walks between the two black bars, increased total walking time, decreased number of breaks and increased walking bout distance	Increase of physical persistence and daily activity
DeAndrade et al. (24)	BTBD9 knockout mouse	Iron depletion in dopaminergic neurons (23)	Total distance in the open field chamber and during wheel running	Mutated mice showed increased total distance during both experiments and were more active during dark periods of the day	Change of physical activity during the day and especially at night

## Main Text

### Iron in PD

Several post mortem and imaging (MRI and ultrasound) studies have shown that iron concentration is an important susceptibility factor for nigrostriatal degeneration and that basal ganglia iron concentration is higher in PD patients compared to controls (27, 28). Increased echogenicity of the substantia nigra (SN) on ultrasound is a typical sonographic finding in PD, and sonographic signal intensity of the SN is related to tissue iron content with higher iron levels being associated with increased echogenicity in both animal models and post mortem findings in human (29–31). Recent studies showed that mutant and wild-type alpha-synuclein may have differential interaction with iron and mutant alpha-synuclein toxicity could be preferentially exacerbated by iron. Iron overload could selectively influence mutant alpha-synuclein toxicity and disease phenotypes (32). Overall, the iron content is elevated in the SN of PD patients (33, 34). A conservative repositioning strategy of iron in an animal model using the iron chelator desferrioxamine retarded 6-hydroxydopamine-induced degeneration of nigrostriatal dopamine neurons (35). In PD application of deferiprone improved the motor part of the Unified PD rating scale (25).

### Iron and Dopamine in PD

Although brain iron pathways and their relevance to PD are not entirely clarified, it is suggestive that iron is important in PD pathophysiology (25, 36). Iron is a significant co-factor in dopamine biosynthesis and is intricately involved in the regulation of dopamine levels in the brain. Several pathways are involved in dopamine metabolism. Iron concentration in neurons is high so that toxic dopamine metabolites occur (37).

In neurons a high iron content is detected in the cytoplasm. In this cellular compartment the formation of toxic dopamine metabolites takes place (38, 39). Iron accumulation is found in several brain regions in synucleinopathies whereat only specific regions are vulnerable for neuronal loss (40). Factors triggering neuronal loss associated are still poorly understood. Neuromelanin is produced in the substantia nigra, pars compacta SNc whereas dopamine is converted to o-quinones (41). Neurotoxic intermediates may result from iron mediated oxidation reactions. The interaction of iron and dopamine has been suggested as major player in the development of neuronal cell loss in defined brain regions (i.e., SNc).I. Neuromelanin as intracellular iron storage is of particular importance in PD (42). Accumulation and overload of iron suggested to increase oxidative stress has be demonstrated in the diseased PD brain (43, 44). The direct interaction of neuromelanin with the protein alpha-synuclein has also been accused to trigger neuronal damage. Alpha-synuclein gene dosages are higher in individual melanized neurons, triggering cell death to a greater extent in PD compared the healthy controls (45–47). Finally, microglia might be activated by neuromelanin leading to an outspread of neuronal degeneration in brain tissue (48).

### Iron in RLS

RLS is the most frequent movement disorder with potentially severe disease burden due to physical and mental health. The clinical key features include sensory symptoms (restlessness, urge to move), unpleasant sensations (paresthesia, pain), and motor symptoms (periodic limb movements, further motor manifestations) (49). Low brain iron (reflected by, e.g., ferritin) despite normal peripheral iron plays a major role through its effects on the dopaminergic system (26, 50, 51). Cerebrospinal fluid (CSF) and serum ferritin are positively correlated. Low CSF ferritin is accompanied by low serum ferritin, particularly in cases diagnosed with iron deficiency anemia. RLS prevalence, as expected, is about five times more prevalent in patients with iron deficiency anemia than in the general population (52, 53). Iron deficiency anemia may predispose to the development of RLS but is no precondition as about 5% of the general population are affected from RLS (53). Beyond iron deficiency itself, the interaction of low CSF ferritin with other secondary biochemical changes and genetic factors lead to RLS symptoms. Iron replacement therapy shows inconsistent results so that general guidelines for treatment do not unambiguously commit to iron treatment (54, 55). Iron deficiency in brain may be related to a loss of iron regulatory protein activity, the function of blood-brain barrier, where endothelial cells act as iron reservoir for the brain. A dysfunction of iron regulatory proteins in the microvasculature might lead to a decrease of iron storage in endothelial cells.

Most probably, RLS is determined by environmental factors and genetical modifiers of brain iron homeostasis leading to a dysfunction of the cortico-striatal-thalamic-cortical circuit.

### Iron and Dopamine in RLS

Using a post-weaning, diet-induced iron deficiency (ID) condition in rodents to reduce SN iron, studies have demonstrated that iron deficiency increases extracellular striatal dopamine, decreases striatal dopamine-2 receptor density, and diminishes dopamine transporter density and function *in vitro* but not dopamine release (56). Similar to the findings in the iron deficiency rodent, RLS striatal dopaminergic pathology has shown diminished dopamine-2 receptors (56). Iron deficiency in rodents lead to decreased ventral midbrain iron concentrations and to changes in the dopamine system that mimic many of the dopaminergic changes seen in RLS patient where low SN iron is a known pathology of the disease (56). It is well-known that RLS occurs with increased dopamine in the striatum (57, 58). Adding iron to the ventral midbrain leads to increased extra-cellular dopamine in the striatum (56). Adding iron to the striatum had no effect on its dopamine levels. Thus, the increase in iron seen in ventral midbrain with intravenous iron treatment seems to induce changes in striatal dopamine seen in RLS itself. Biochemical studies on the effect of iron in brain indicated that iron regulates hypoxia inducible co-factor pathways, leading to an upregulation of proteins that are involved in angiogenesis, erythropoiesis, cell survival, and proliferation. The interplay between compromised neuronal iron uptake and the functions of the neuromelanin-containing and dopaminergic cells might lead to RLS pathology (59). Decreased density of dopamine-1 and dopamine-2 receptors are detected in conditions of iron deficiency, indicating that iron affects the brain dopaminergic transmission in different ways (60). In RLS it has additionally been reported that A 10 and A11 dopaminergic neurons, localized in the hypothalamus, might be involved in disease mechanism (1). It has been suggested that these neurons are connected to the spinal cord in mice modulating spinal excitability leading to alterations. These changes lead to disturbed sensory processing of leg afferents projecting in brain stem structures (2).

Iron dyshomeostasis in SN is characteristic for both PD and RLS. The misdistribution of iron leads to iron deposits in PD and iron deficiency in RLS, presenting mechanistically a “relatively inverse” picture between PD and RLS. However, the picture may be much more complex because the misdistribution can also lead to both deposits and deficiency at the level of cell compartments (e.g., mitochondria vs. cytoplasma), cells (neurons vs. astrocytes and microglia) and tissue (e.g., SN vs. striatum and other regions). The complex picture can explain why some patients present both PD and RLS (61).

## Animal Models for Movement Disorders Associated With Iron Metabolism

Based on the assumption that animal models associated with iron dyshomeostasis in the brain, more specifically in the basal ganglia, may have the potential to report about mobility and movement patterns that may also be altered by humans affected by the “corresponding” disease, we searched PubMed for studies investing such animal models and including quantitative data on mobility and movement patterns, and have been published until September 2018 (Tables 1, 2). We used the following search terms: “animal + iron,” “Parkinson disease iron animal model,” “Parkinson disease iron mouse model,” “Parkinson disease iron rat model,” “Parkinson disease iron drosophila model,” “Parkinson disease iron activity levels mouse,” “Iron dopaminergic system mouse model,” “iron metabolism mouse model,” “Restless legs animal model,” “Restless legs iron mouse model,” “Restless legs iron rat model,” “Restless legs iron drosophila model,” and “Restless legs iron activity levels mouse.” Articles were then checked for duplicates and for fulfilling the inclusion criteria (animal model, direct/indirect evidence of altered iron content in the basal ganglia, quantitative movement, and mobility assessment). We identified nine studies whereof six studies fulfilled these inclusion criteria and were thus included in the final evaluation (Tables 1, 2). Four studies investigated models with iron overload (15, 17, 20, 21) and two studies investigated models with iron deficiency (23, 24). All but two [drosophila melanogaster (20, 23)] were mouse models. Two studies used the identical mouse mutant (15, 21). The six studies are presented in following in more detail.

## Motor Behavior in Animal Models With Elevated Brain Iron Content

Old neuroferritinopathy mutant mice, which have increased accumulation of iron aggregates in the striatum among other brain regions, showed a shorter latency to fall from the rotarod compared to WT mice (21) (Table 1). These mutant mice presented with a progressive behavioral and motor phenotype, consisting of abnormal grooming, tremor, posture, and incoordination (21). Feet clasping was observed after suspension of the mice by their tails. Moreover, they flexed their front and hindlimbs inward, with paws clasped together and drawn in toward the body. In contrast, WT mice demonstrated normal limb posture when suspended by their tails. Toward the end of the life span, mutant mice displayed a hunchbacked posture when walking and low body position with some degree of lordosis. They also showed a shuffling gait with dragging hindlimbs, suggestive of a reduction of position reflexes. In these mice, iron was elevated in multiple brain regions but highly accumulated in the dorsal striatum that is in humans known to mediate motor and executive functions (e.g., inhibitory control). The connection between the location of the damage and observed mobility deficits possess a degree of similarity to motor symptoms occurring in PD.

In another study using the same transgenic mice, the analysis of motor performance on a rotating rod revealed a significant decrease in performance at the earliest age tested (4 months), before an obvious clinical phenotype was observed (Table 1). This effect was somewhat stronger after 13 months of age, suggesting indirect iron-dependent disturbances in dynamic balance (15). Accordingly, rise of iron deposits within a brain region and across regions was positively associated with disease progression in PD patients (62). Taken together, the observed changes in mobility and movement performance of this mutant mouse strain reflect dynamic balance disturbance and motor coordination, reduced position reflexes, postural deficits, and reduced physical activity. Of note, the best surrogate parameter currently known for dynamic balance is gait variability, which deterioration is associated with increased frequency of falls in PD (63). This parameter (63, 64) as well as physical activity (65, 66) can be measured with high accuracy using mobile technology.

In a study aiming to evaluate the role of dietary iron restriction in a mouse model of PD (Table 1), mice were fed diets containing low (Fe-, 4 ppm) or adequate (Fe+, 48 ppm) amounts of iron for 6 weeks before the administration of 1-methyl-4-phenyl-1,2,3,6-tetrahydropyridine (MPTP) (17). MPTP is a mitochondrial toxin that damages nigrostriatal dopaminergic neurons and induces PD-like symptoms. Administration of MPTP resulted in reduced levels of dopamine and its metabolites in the striatum of Fe+ mice but not Fe- mice. Consistently, MPTP impaired postural control as determined by the mean number of falls from the rotarod and total time spent on the rotarod only in Fe+ mice, but not Fe– mice (17). Iron therefore seems to favor formation of cell death in face of other factors damaging dopaminergic neurons. In conclusion, this study suggests that even in non-mutant animal models reflecting (at least some aspects of) PD, iron homeostasis influences the motor phenotype -more specifically dynamic balance- and that dietary iron restriction has a beneficial effect.

Another study used a drosophila melanogaster model overexpressing A53T or A30P alpha-synuclein with (Fe–) and without dietary iron restriction (Fe+) (Table 1) (20). The authors found an iron overload in dopaminergic neurons only in mutated Fe+ animals but not in all other groups (including the mutated Fe– group). The mutated flies without iron restriction also performed worse in a negative geotaxis assay (a plastic column where the flies had to climb above a certain line), suggestive of reduced motor activity and/or reduced voluntary movements. This study also demonstrates that iron overload, in combination with additional factors (here PD-modulating genetic mutations), can promote degeneration of dopaminergic neurons. These results fit well-with the above observations of reduced physical activity, although based on these results it is difficult to exclude potentially contributing symptoms such as apathy and fatigue.

## Motor Behavior in Animal Models With Reduced Brain Iron Content

In *BTBD9* knockout mice, a RLS model, physical activity levels, sensory perception, sleep architecture were determined (Table 2) (24). Mutant mice showed a significant increase in physical activity compared with WT mice. Interestingly, the mutant mice did not show sensory deficits during the middle of the night (the active phase) but during the middle of the day (the rest phase), suggesting an alteration of the circadian rhythm. To analyze sleep architecture, the mice were implanted with a wireless telemetry system. Mutated mice showed decreased overall sleep time but no change in the amount of rapid eye movement sleep (24). Although the pathophysiological aspects of the above-mentioned association are, to our best knowledge, not yet understood, these mobility, and movement alterations are interesting in the light of mobile technology applied to men. This novel technology is able to evaluate physical activity and sleep performance such as movements during sleep (67).

In a RLS drosophila melanogaster model with loss of functional *BTBD9*, increased locomotor activity was found in a camera-based assessment of total walking distance, number of walks, total walking time, number of breaks, and average walking bout distance (Table 2) (23). Moreover, the mutated animal model presented with increased sleep fragmentation, including shorter, and more frequent sleeping bouts. The authors hypothesized that these phenomenon are due to the mediation of different sleep and activity phenotypes by *BTBD9* (via the alteration of neurotransmission in some dopaminergic neurons (23). Whatever the reasons for the observed phenotypes are, mobile technology can readily and accurately assess such movement patterns in humans (68).

## Conclusion

Although we tried to comprehensively analyze/discuss the articles according to our hypothesis, a not negligible limitation of our review is that we could only include six studies to our analysis which fulfilled the inclusion criteria. We tried to set the review in perspective with the state-of-the art methodology in analyzing the influence of iron on mobility in animal models.

**Figure 1:**
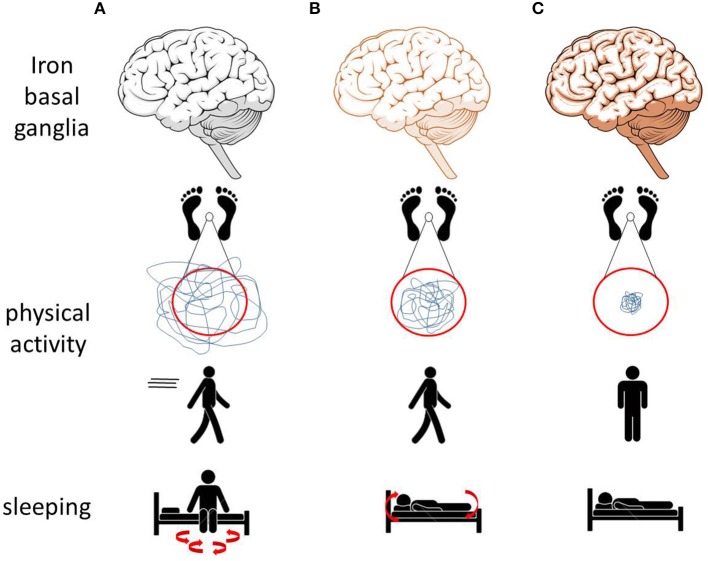
Effects of altered brain (especially basal ganglia) iron homeostasis on movements and mobility observed in animal models may translate to similar alterations in men. **(A)** Low iron levels in animal models are associated with increased physical activity and with sleep fragmentation. **(B)** Normal iron levels are associated with normal physical activity, normal sleep behavior, and the ability to move during sleep. **(C)** Increased iron levels are associated with reduced physical activity, postural and trunk instability, and immobility while sleeping (69, 70).

Our systematic evaluation of the currently available peer-reviewed studies assessing movement and mobility in animal models with altered brain iron suggests that elevated basal ganglia iron leads to a decrease in motor performance, particularly affecting postural, and trunk stability (Figure 1). Low basal ganglia iron levels were mainly associated with increased physical activity, mobility, and sleep fragmentation as well as with changes of the circadian rhythm (Figure 1). The above-mentioned mobility and movement parameters may be particularly promising to assess effects of conservative iron repositioning on movement and mobility in PD.

## Author Contributions

FH, MH, WM, MP, and DB drafting and revision of manuscript, Fair-Park II study group. FH, WM, DD, CH, CMa, and CMo study concept and design. FH, MH, WM, DD, CH, and CMo interpretation of data.

### Conflict of Interest Statement

The authors declare that the research was conducted in the absence of any commercial or financial relationships that could be construed as a potential conflict of interest.

## Abbreviations

PD: Parkinson's disease
RLS: restless legs syndrome
ID: iron deficiency
CSF: Cerebrospinal fluid
SN: substantia nigra
MPTP: 1-methyl-4-phenyl-1,2,3,6-tetrahydropyridine.
